# Preparedness of Dentists to Manage Anxiety in Developmentally Disabled Patients

**DOI:** 10.1155/2023/1903411

**Published:** 2023-09-08

**Authors:** Maura B. Lynch, Lynn M. Tepper, Steven Chussid, Renuka Bijoor

**Affiliations:** ^1^College of Dental Medicine, Columbia University, New York, NY, USA; ^2^College of Dental Medicine and School of Public Health, Columbia University, New York, NY, USA; ^3^Division of Pediatric Dentistry, College of Dental Medicine, Columbia University, New York, NY, USA

## Abstract

**Background:**

Recently, the National Council on Disability urged the Commission on Dental Accreditation to require more thorough training in the care of developmentally disabled patients. Curricula is early in its development and its' effectiveness is unknown.

**Objectives:**

The aim of this study was to determine if special needs dental education has had an impact on providers' professional behavior, practice characteristics, attitudes, and confidence when treating these patients and managing their dental anxiety.

**Methods:**

A nonrandomized, noninterventional, and anonymous, Qualtrics survey was administered prospectively to members of one local and one national organization.

**Results:**

Of the 107 respondents (response rate of 81.06%), 89% treat special needs patients. Positive reinforcement (88.64%), distraction (85.23%), and enhancing control (85.23%) were the modalities most used by these participants. Regarding treating this population, the average response regarding both confidence and wanting to learn more fell between disagree and agree at 2.92, while the average response regarding concern about safety fell between agree and strongly agree at 3.22. Level of specific expertize had the strongest influence on a provider's decision to treat, while reimbursement level had the least influence. Among participants who completed postdoctoral education, the average response for how well their education prepared them to manage patients with special needs was higher for their postdoctoral training compared to their dental school training, with pediatric dentists reporting the highest rate of preparation. No correlation was found between year of dental school graduation and how well they felt their education prepared them for treating this population. Significance level was set at 5%.

**Conclusion:**

Dental education can have a significant impact on dentists' knowledge, attitudes, beliefs, and confidence about treating those with developmental disabilities and managing their dental anxiety. Acknowledging that this relationship exists should encourage educational improvements in this area.

## 1. Introduction

In the American study, one in four US adults have disabilities that challenge them on a daily basis [1]. Given this ratio of individuals with special needs in the United States, it is crucial that they have access to dentists who are willing and able to treat them. Treating patients with disabilities requires a particular skill set that must be developed over time through thorough training. It is the responsibility of dental educators to provide their students with the knowledge and experience that is required to serve these patients and their unique dental needs. Time and effort must be put toward in both creating a curriculum that satisfies these requirements and developing a means to assess the efficacy of the education offered in this area. Our research questions assessed the effectiveness of dental education in preparing dental professionals to treat this vulnerable population.

The Commission on Dental Accreditation of the Dental Association has recently implemented a new standard in the education of students in treating those with the intellectual and physical disabilities. This came in response to the National Council on Disability putting pressure on the commission to require dental schools to provide the students with more thorough training in treating this population [2]. Complete and comprehensive curricula is therefore early in its development and the effectiveness of it is unknown.

The aim of this study was to determine if special needs dental education, or the lack thereof, has had an impact on providers' professional behavior, practice characteristics, attitudes, and confidence when treating these patients and managing their dental anxiety. Answering this question will provide feedback on the influence dental education has on the quality of care provider's are able to deliver to this population. Results demonstrating that dental education has an impact, could motivate dental educators to focus their attention on this important area of the curriculum. The results demonstrating that dental education does not have an impact, could inspire researchers to discover what it is that contributes to providers' professional behavior, practice characteristics, attitudes, and confidence when treating patients with special needs and managing their dental anxiety. Either way, the results will allow the focus to be directed toward modifying factors that have the potential to make a significant difference. With this in mind, the current study was created to address the importance of preparing dentists to treat this population in effort to improve the dental education in this area.

## 2. Materials and Methods

The study design was a nonrandomized, noninterventional, and anonymous survey using Qualtrics. To ensure validity and reliability, the questions were modified from a previous study [3] and the methods description partly reproduces their wording. Request for access to this previous questionnaire was approved by the author. The current study's 26-item online questionnaire was created through Qualtrics, and responses were recorded between September 21, 2020 and December 8, 2020. It was administered prospectively to the 107 respondents who met the inclusion criteria of being dental practitioners who completed their dental education programs. It was distributed via two dental practitioner organizations—(1) emailed to a list of Tristate New York, New Jersey, and Connecticut Dental Care practitioners and (2) posted on a Facebook-based discussion forum of dentists. Participants completed the survey remotely and the sample included 69% general dentists, 19% pediatric dentists, 4% oral surgeons, 3% periodontists, 2% orthodontists, 2% prosthodontists, and 1% endodontists.

The survey asked questions concerning which categories of adult and pediatric special needs patients the dentists treat, if any; which anxiety interventions they provide for these patients; and which special arrangements they make to be able to treat these patients. Questions concerning the dentists' demographic information, educational background (dental and predental), personal experiences, and attitudes concerning the treatment of special needs patients were included as well. Established interventions used to manage the dental anxiety were incorporated into the survey [4]. The data were collected anonymously through Qualtrics and analyzed on Microsoft Excel.

## 3. Results

**Figure 1:**
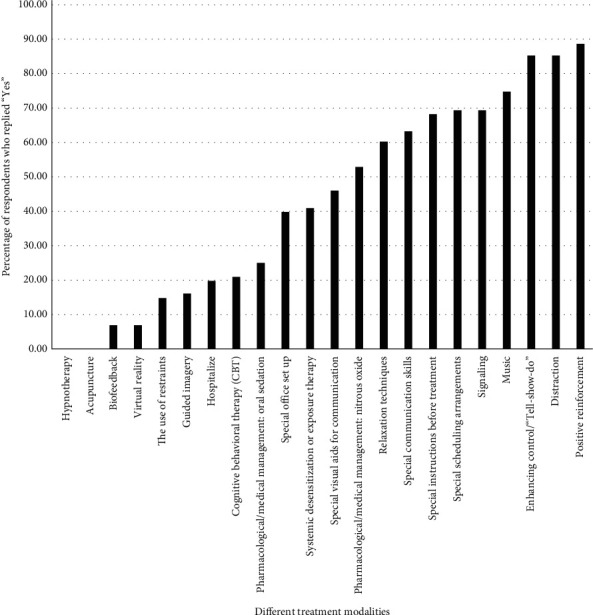
Percent of respondents who replied “Yes” to utilizing the following treatment modalities when treating their special needs patients.

Of the 107 respondents (response rate of 81.06%), 89% reported that they do treat special needs patients including those who have mobility/movement impairments, hearing impairments, vision impairments, mental challenges, autism spectrum disorder, cerebral palsy, neurological disorders, and/or movement disorders (Parkinson's). Positive reinforcement (88.64%), distraction (85.23%), and enhancing control (85.23%) were the modalities most used by these participants. Virtual reality (6.90%) and biofeedback (6.90%) were the modalities least used, and no participants used acupuncture or hypnotherapy (Figure 1).

**Figure 2:**
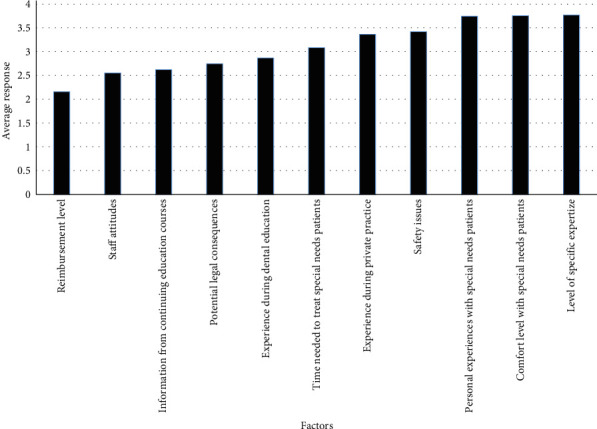
Factors influencing your decision to treat special needs patients. 1 = not at all influential; 2 = slightly influential; 3 = somewhat influential; 4 = very influential; 5 = extremely influential.

**Figure 3:**
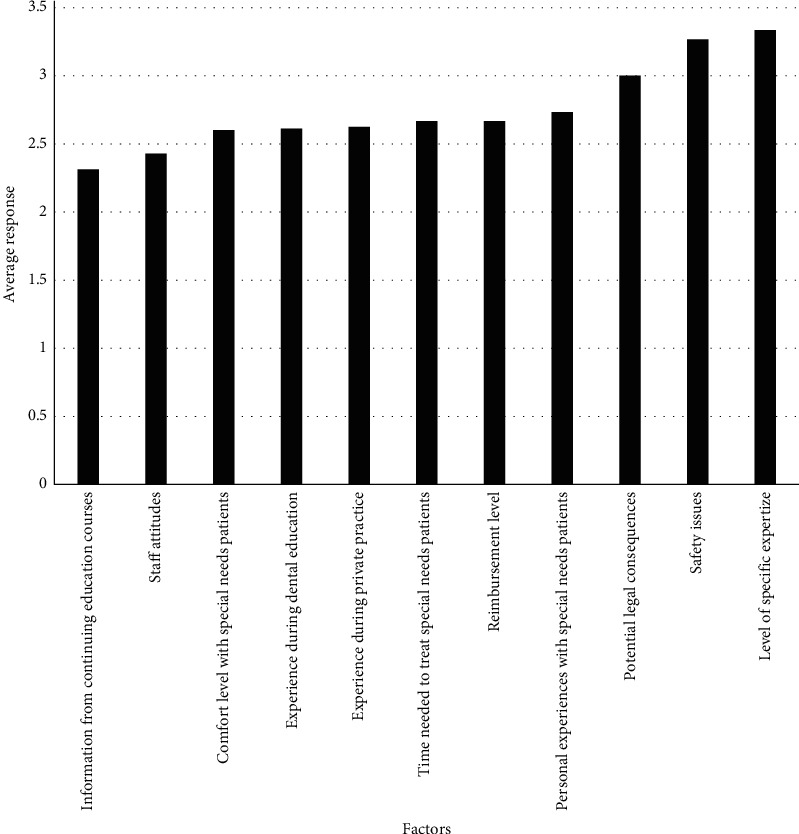
Factors influencing your decision not to treat special needs patients. 1 = not at all influential; 2 = slightly influential; 3 = somewhat influential; 4 = very influential; 5 = extremely influential.

Of those surveyed, the average response regarding comfort level prescribing oral sedation fell between strongly disagree and disagree at 1.65, the average response regarding both confidence in treating this population and wanting to learn more about treating this population fell between disagree and agree at 2.92, and the average response regarding concern about the safety of treating this population fell between agree and strongly agree at 3.22 (Table 1). Level of specific expertize had the strongest influence on a provider's decision to treat or not to treat the special needs patients with an average response falling between somewhat influential and very influential at 3.77 for those who do treat (Figure 2) and at 3.33 for those who do not treat (Figure 3). Participants who do treat this population reported reimbursement level as having the least influence on their decision to treat with an average response of 2.16, falling between slightly influential and somewhat influential (Figure 2). Participants who do not treat this population reported information from continuing education courses as having the least influence on their decision not to treat with an average response falling between slightly influential and somewhat influential at 2.31 (Figure 3).

When asked how well their education prepared them for managing the patients with special needs on a scale of 1 being “not at all” and 4 being “very well”, the average response among participants who received postdoctoral education in a dental specialty was lower for their dental school education at 2.05 and higher for their postdoctoral training at 2.97. Pediatric dentists reported the highest rate of preparation with an average response of 3.81 to this same question regarding their postdoctoral training. No correlation was found between year of dental school graduation and how well they felt their dental school education prepared them for treating this population.

## 4. Discussion

Acknowledging that dental education has the potential to alter dentists' knowledge, attitudes, beliefs, and confidence about treating those with developmental disabilities and managing their dental anxiety can have a significant impact on addressing the importance of preparing dentists to treat this population.

Dental education in this area has been a topic of discussion for many years. In 1957, dental care for handicapped children was only a relatively recent addition to dental school curriculums [5]. Since then, curriculum guidelines have been slowly modified regarding treating this population. In 1979, predoctoral dental training guidelines in the treatment of patients with handicapped conditions were published in the Journal of Dental Education [6]. In 1985, curriculum guidelines for dental students treating patients with minor disabling conditions [7] and for general practice residents treating patients with developmentally disabling conditions were published [8]. Beginning on July 1, 2021, the Commission on Dental Accreditation required all US predoctoral dental education programs to train their students to treat patients with intellectual and developmental disabilities [9]. It is important to recognize that even with these curriculum modifications that have taken place over the years, dentists' knowledge, attitudes, beliefs, and confidence about treating those with developmental disabilities and managing their dental anxiety can still be improved. For instance, there are many treatment modalities, such as virtual reality, that are being utilized much less frequently than modalities such as positive reinforcement and distraction which according to the current study and existing literature are some of the most common modalities used. This may be due to challenges the dental field is facing in implementing virtual reality in both the clinical setting with patients and the educational setting with students such as insufficient evidence regarding ethical aspects, high costs, and complex operability of the systems [10]. Furthermore, practitioners may opt to use these more common tools for a variety of reasons including high-parental acceptance of the use of these methods [11] and perhaps they require less skill and training than other methods. For lack of skills and training in behavior management strategies can present a barrier to care and thus their application must be evidenced based and rooted in sound clinical principles [12]. Due to lack of education in many of these modalities, dentists may need to be supported in their understanding and application of these methods, but many dentists do not have access to the interprofessional collaboration that is needed, especially in the private practice setting.

Dental education can and needs to be improved in this area for patients with special needs have more unmet dental needs compared to the general population [13]. Although 89% of the current study's participants reported that they do treat special needs patients, this majority is not replicated in many populations. For example, in previous years, Oakland County in the state of Michigan has had 882 general dentists and 180 dental specialists, but only 36 dentists were willing to treat the patients with special needs [14]. A survey sent to students at six special education schools in this county found that the most limiting barrier to dental care access for these special needs patients was finding a dentist willing to treat them and this finding has been consistent in other states as well [14].

This lack of willingness of dentists to treat special needs patients is related to the lack of continuum of care when these patients transition from seeing pediatric dentists to seeing general dentists [15]. In this current study, pediatric dentists reported the highest rate of preparation which is consistent with the literature for their 2 years of additional training provides them with the skills necessary to care for children and youth with special health care needs [16]. However, in the United States, there are approximately 7,600 practicing pediatric dentists which is not adequate to care for all these patients [16]. About 70% of pediatric dentists who responded to a survey conducted in 2009 reported that the main barrier for this transition from pediatric to general dentist offices was the lack of general dentists willing to treat this population [14].

In the current study, the average response regarding both confidence in treating special needs patients and wanting to learn more about treating special needs patients fell between disagree and agree. Much of the literature on this topic claims that the lack of confidence general dentists have in treating this population is a reflection of the inadequate education they receive in dental school on this topic [14]. Furthermore, the participants in the current study who do no treat special needs patients reported continuing education as having the least influence on their decision not to treat. This could be an area for potential improvement moving forward as continuing education courses put limited emphasis on special care dentistry but requiring credits in this area could increase education and awareness among general dentists on the access to care issue that exists [14].

There are limitations to the current study that must be considered. Participants overall perception of their dental education may have influenced their responses and is therefore a potential limitation to this study. It is difficult to distinguish whether it was a participant's actual education or their perception of their education that made them respond to the questions in one way or the other. For example, a respondent may have reported feeling underprepared to treat this population not because the education in this area was lacking, but because they had a negative experience with their dental school in general. Another potential limitation is our sample. The majority of the sample was made up of general dentists and pediatric dentists. Future studies can aim to address participants' perceptions of their dental education in general and include a more diverse sample of dental specialists. It would also be helpful to administer a similar questionnaire when today's current dental students and future dental students graduate after receiving an education with the recently implemented curriculum modifications regarding treating the special needs patients. The results from our current study could be compared to this future study to better assess the effectiveness of these new curriculum guidelines in order to continue to improve the dental education in this area.

## 5. Conclusion

There is an imperative need to improve both predoctoral and postdoctoral education in this area for dental education can have a significant impact on dentists' knowledge, attitudes, beliefs, and confidence about treating those with developmental disabilities and managing their dental anxiety. The findings from this study have significant implications for dental education. As mentioned previously, one of the main barriers to access to care for special needs patients is finding dentists who are willing to treat them. This challenge will continue to be exacerbated by the fact that the life expectancy of developmentally disabled patients is increasing with advancements in health care and therefore, the number of these patients who need dental treatment will continue to grow. With increasing demand, it will no longer be sufficient to solely rely on the pediatric dentists to treat those with developmental disabilities thus, other dental providers must be willing to treat this population. However, the treatment of these patients has become so complex that it has led to the evolution of special care dentistry defined as “a method of oral health management that is specially designed for patients with special needs who have a variety of medical conditions or disabilities that require more time or altered delivery methods than the routine delivery of dental care for the general population [17].” Thus, dental education must be enhanced to address this complexity for a dental practitioner's willingness to treat this population is hindered by their perceived level of preparedness [18, 19]. This change must happen now, for the fewer providers there are willing to treat special needs patients today, the fewer providers there will be who are prepared to educate future dental students to be competent in treating this population, causing these issues to persist [20, 21]. By training more students to properly treat this population via an improved dental education curriculum, we will increase these individuals access to care and thus improve the treatment and management of dental anxiety in this vulnerable population.

## Figures and Tables

**Table 1:** Respondents' self-reported attitudes and behaviors toward treating special needs patients.

Statements about treating special needs patients	Average response
I feel comfortable prescribing oral sedation for special needs patients	1.65
I feel comfortable using physical restraints on special needs patients	1.68
Financial compensation for treating special needs patients is adequate	2.01
My dental education prepared me well for treating special needs patients	2.42
I have seen an increase in the number of special needs patients over the years in my practice	2.61
My staff is knowledgeable in treating special needs patients	2.67
My practice is set up for the treatment of special needs patients	2.67
I am concerned about legalities when treating special needs patients	2.72
My staff is comfortable treating special needs patients	2.82
I like to treat special needs patients	2.90
I am confident treating special needs patients	2.92
I would like to learn more about treating special needs patients	2.92
I am concerned about the safety of special needs patients	3.22

*Note*: A mean value of 1.65 demonstrates that the calculated average response to that statement fell between 1 = strongly disagree and 2 = disagree. 0 = Not applicable; 1 = strongly disagree; 2 = disagree; 3 = agree; 4 = strongly agree.

## Data Availability

Data are available on request by contacting the primary author of this study, Maura Lynch, by phone (708-638-6054) or by email (mlynch0817@gmail.com).
